# Hydrogel Delivery Systems for Biological Active Substances: Properties and the Role of HPMC as a Carrier

**DOI:** 10.3390/molecules30061354

**Published:** 2025-03-18

**Authors:** Arailym Amanzholkyzy, Shynar Zhumagaliyeva, Nurgul Sultanova, Zharylkasyn Abilov, Damira Ongalbek, Elvira Donbayeva, Aktoty Niyazbekova, Zhazira Mukazhanova

**Affiliations:** 1 Department of Chemistry and Chemical Technology, Al-Farabi Kazakh National University, Al-Farabi Ave. 71, Almaty 050040, Kazakhstan; arai13_95@list.ru (A.A.);; 2 Department of Chemistry, L.N. Gumilyov Eurasian National University, Astana 010000, Kazakhstan; 3 Department of Veterinary Science and Technosphere Safety, West Kazakhstan Innovation and Technological University, Uralsk 090009, Kazakhstan; 4 Higher School of IT and Natural Sciences, S. Amanzholov East Kazakhstan University, Ust-Kamenogorsk 070010, Kazakhstan

**Keywords:** hydrogels, plant extracts, cellulose derivatives, cellulose ethers, drug delivery, release, HPMC, dosage forms, films

## Abstract

Hydrogel delivery systems are popular dosage forms that have a number of advantages, such as ease of use, painlessness, increased efficiency due to prolongation of rheological, swelling and sorption characteristics, regulation of drug release, and stimulus sensitivity. Particular interest is shown in hydrogels of cellulose ether derivatives due to the possibility of obtaining their modified forms to vary the solubility, the degree of prolonged action, and the release of the active substance, as well as their widespread availability, affordability, and the possibility of sourcing raw materials from different sources. Hydroxypropyl methylcellulose (HPMC, “hypromellose”) is one of the most popular cellulose ethers in the production of medicines as a filler, coating and carrier. Research on hydrogel carriers based on polymer complexes and modified forms of HPMC using acrylic, citric, and lactic acids, PVP, chitosan, Na-CMC, and gelatin is of particular interest, as they provide the necessary rheological and swelling characteristics. There is growing interest in medical transdermal hydrogels, films, capsules, membranes, nanocrystals, and nanofibers based on HPMC with the incorporation of biologically active substances (BASs), especially those of plant origin, as antibacterial, wound-healing, antimicrobial, mucoadhesive, anti-inflammatory, and antioxidant agents. The aim of this article is to review modern research and achievements in the field of hydrogel systems based on cellulose ethers, particularly HPMC, analyzing their properties, methods of production, and prospects for application in medicine and pharmacy.

## 1. Introduction

In the field of development of medical drugs and products, among all popular forms of drug delivery, hydrogel delivery systems have a number of advantages. Hydrogel-based drug delivery systems are easy to use, painless, and offer enhanced efficiency due to the prolonged release of the active ingredient. An important distinction of hydrogel systems is their convenience in use. This difference allows them to be considered an alternative to traditional forms. By selecting and combining an active ingredient with a hydrogel base, transdermal systems that have antiviral, antibacterial, and local anesthetic effects are developed [1,2,3,4]. Hydrogels also have the ability to respond to changes in external conditions, such as pH, temperature, electric field, light, ionic strength, magnetic oscillations, etc., which allows them to be classified as intelligent materials with adjustable properties. Additional properties of hydrogels include biodegradability, biocompatibility, and sensitivity to various factors. Biodegradability is currently [1,5,6] an important property for the development of environmentally friendly materials and technologies.

Hydrogels, which are three-dimensional polymer networks crosslinked through physical and chemical interactions, are synthesized from polymers of synthetic origin, semi-synthetic, and natural origin [7,8,9]. Polymers of different origins have both advantages and disadvantages. Synthetic and semi-synthetic polymers, while possessing the ability to polymerize and excellent mechanical properties, cannot surpass natural ones in terms of biocompatibility with the human body, and are also not capable of creating an accessible environment for other cells. Among the wide range of natural polymers, including those of protein, nucleic acid, and polysaccharide origins, this review will focus on cellulose ether derivatives, in particular, HPMC and hydrogels based on them [10,11]. As recent literature reviews show, cellulose derivatives are among the leading materials for pharmaceutical hydrogel production due to their widespread availability in nature, non-toxicity, and low cost.

Hydrogel systems are capable of swelling in water and aqueous solutions, which makes them compatible with the human body. Hydrogels also function as matrices in which biologically active substances can be immobilized, including synthetic and natural compounds, and plant extracts, for biomedical applications and enhancing biocompatibility [7]. Especially promising “green” sources of biologically active substances are plants rich in phenolic compounds, flavonoids, tannins, and others. In addition to biocompatibility and safety, another important property of hydrogels is their ability to prolong the action of a drug. This is particularly significant when developing medications for long-term use and high-toxicity treatments, in cancer therapy, and in the treatment of slow-healing wounds using pain-relieving agents. For instance, studies [12,13,14] have reported the development of drug formulations containing lidocaine and prilocaine microemulsions, which are local anesthetics with prolonged action, targeted delivery, and enhanced drug efficacy, demonstrating the promise of research in this direction. The potential of using cellulose derivatives as drug carriers, mathematical models of drug release, and the feasibility of creating ointment and gel drug formulations with prolonged action have also been demonstrated in [15,16,17,18,19,20]. Previously, the authors of [21,22,23] obtained hydrogels of the anesthetic substances rihlocaine and AK-29. New hydrogel dressings were developed using the radical crosslinking method based on poly-N-vinylpyrrolidone. These dressings contain the plant-derived medicinal substance “Alhidin”, extracted from the plant camelthorn (*Alhagi kirghisorum Shrenk.*), which grows in Kazakhstan [24]. The authors of [25] also obtained extracts from the plant *Tamarix* of the *Tamaricaceae* family, which were subsequently immobilized into polymer matrices in the form of films [26]. Previously, polyphenolic, terpenoid and other biologically active compounds from plants of this genus were found and characterized. Extracts and individual components have shown antioxidant, anti-amnesic, antimicrobial, antifungal, and cytotoxic activity. Using extracts of *Tamarix Hispida* as the aqueous drug component of HPMC hydrogel, we can obtain hydrogels with the abovementioned properties and use these widely in medicine and pharmacology [27].

The proposed review is a logical continuation of the studies of prolonged systems with biologically active substances based on polymer hydrogels and their compositions of synthetic and natural origin and will be used to study the possibility of creating hydrogel carriers of plant extracts based on HPMC and its compositions.

**Figure 1:**
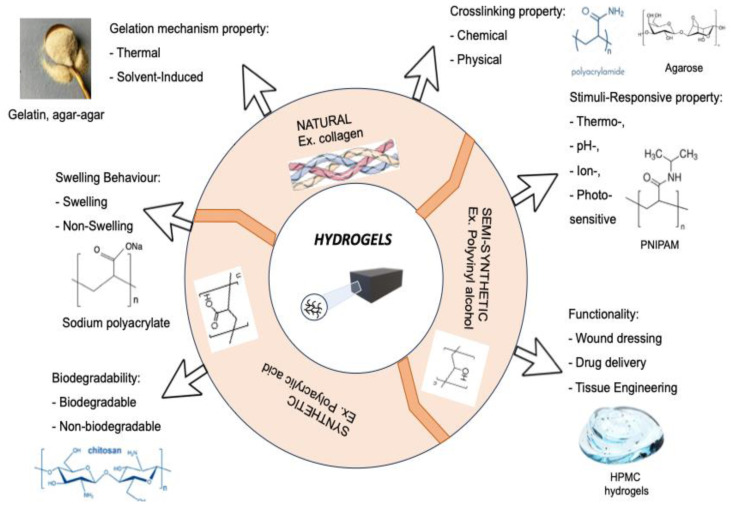
Classification of hydrogels based on their properties [7].

Among natural polymers for medical purposes, cellulose is advantageously distinguished by its diversity due to its modified forms. The introduction of additional groups into the structure of cellulose allows for varying their solubility, and provides the opportunity for immobilization of medicinal substances and regulation of release. As mentioned above, hydrogels are classified into natural, semi-synthetic, and synthetic based on their origin (Figure 1, [7]). The classification of hydrogels is based on their physical and chemical properties, as well as their structural features.

It should be noted that it is hydrogels based on natural polymers that have been put to use in such an important field as medicine. Hydrogels are used in the production of contact lenses, matrices for cell proliferation, bases for targeted drug delivery, stabilizers for nanoparticles, composite materials, and more [28,29].

## 2. Cellulose Ether Derivatives as Hydrogel Bases

**Figure 2:**
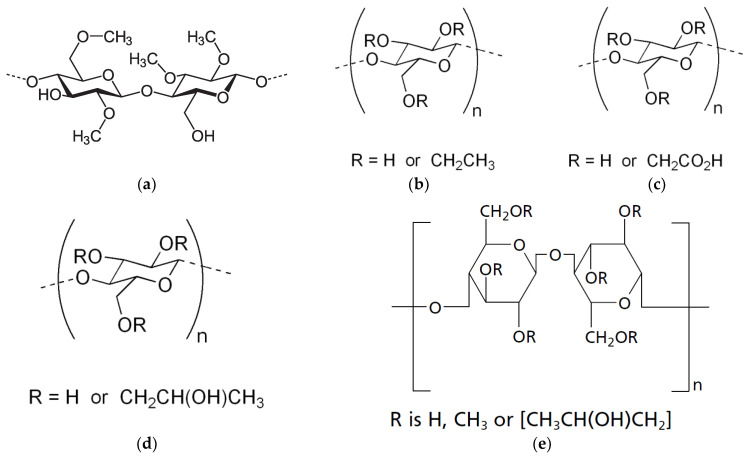
Cellulose ether derivatives methylcellulose (MC) (**a**), ethyl cellulose (EC) (**b**), carboxymethylcellulose (CMC) (**c**), hydroxypropyl cellulose (HPC) (**d**), hydroxypropyl methylcellulose (HPMC) (**e**) [39].

Cellulose and its derivatives, particularly cellulose ethers [30,31,32], are rightfully considered the favorites among natural raw materials for hydrogels. This is evidenced by the studies of various authors [18,19,29,33,34,35,36]. A significant advantage of cellulose derivatives is their abundance, which allows for a wide range of raw materials to be used for their production, such as various grains and cotton [37]. Cellulose derivatives, as complex esters, find widespread application in the pharmaceutical, food, and cosmetic industries. Among cellulose derivatives, the most widely used are methylcellulose (MC), hydroxypropyl cellulose (HPC), hydroxyethyl cellulose (HEC), and hydroxypropyl methylcellulose (HPMC) [38,39] (Figure 2).

Among the cellulose derivatives mentioned above, the most popular in the production of medicines is HPMC, which, in many literary sources, is called “hypromellose”. Hypromellose is a key component of drug coatings and fillers. This cellulose ether has advantages over other derivatives such as solubility in aqueous media, good swelling capacity, polymer viscosity, biocompatibility and biodegradability [40]. Hypromellose, like chitosan, has the advantage of having a complex-forming ability [41,42]. Polymer complexes and the modification of HPMC are also widely used to obtain hydrogel carriers of medicinal substances and materials with the necessary rheological and swelling characteristics. Thus, a number of studies [43,44,45,46,47] are dedicated to the development and study of the properties of hydrogels of cellulose derivatives crosslinked with acrylic acid and the delivery of such medicinal substances as galantamine hydrobromide and benzophenone-4. The structural and rheological properties of the hydrogel matrix play an important role in the development of drug delivery systems. These properties can be regulated by selecting a crosslinking agent, copolymerization, and altering external conditions such as temperature, ionic strength, and pH. To improve the physical, mechanical, rheological and bioadhesive properties, three-dimensional hydrogels with polyvinylpyrrolidone (PVP) were synthesized, which have swelling and water-retaining capacity for transporting medicinal substances.

In general, the processes for producing hydrogels are quite simple. In pharmaceutical production, hydrogels can be used both with and without the inclusion of a medicinal substance. For example, the medications Hitopran (Prontosan), Purilon, Matopat, Granugel are used in medical practice as wound-healing dressings in the form of hydrogel bandages and do not contain an active ingredient. Medical gels based on cellulose additionally include the following components: antimicrobial preservatives (methylparaben, propylparaben, chlorhexidine gluconate), stabilizers (disodium edetate), dispersing agents (alcohol, glycerin, propylene glycol, sorbitol), and homogenizers. The gelling ability depends on the type and molecular weight of the cellulose derivatives, but the minimum gelling concentration ranges from 4% to 6%. The convenience of using cellulose-derivative-based gels lies in their simple preparation, the absence of additional crosslinking agents, and the use of purified water as the hydrophilic phase. The methods for obtaining HPMC hydrogels are based on both traditional and environmentally friendly alternative methods, which increases their applicability.

## 3. Methods for Obtaining Hydrogels Based on HPMC

The successful development of hydrogel-based drug delivery systems is determined by the initial stage of creating the hydrogel matrix. This involves using both traditional methods and, increasingly in recent years, innovative techniques employing more environmentally friendly approaches. The search for new approaches to hydrogel synthesis is driven by the fact that hydrogels produced using traditional methods often exhibit low mechanical stability and slow responsiveness to external conditions [48].

The order of mixing components plays an important role in the preparation of gel formulations. Mixing the components above with the gelling agent should be carried out considering their influence on the gelling process. If these ingredients affect the rate and extent of swelling of the gelling agent, they are mixed after gelling. If such an intervention does not occur, the drug and other additives are mixed before the swelling process; in this case, it is also important to consider the impact of swelling time, mixing temperature, and other processing conditions on the physicochemical stability of the formulation and additives. Typically, the following order of mixing is recommended:

1. The active ingredients are dissolved or suspended in the hydrophilic phase necessary for preparing the gel.
2. Other additives are dissolved in the obtained solution or in a small amount of the hydrophilic phase, accordingly.
3. If necessary, the dispersion of the active ingredient is mixed with the solution of additives.
4. The gelling agent powder is added to the resulting dispersion solution with gentle stirring and left to swell.

When preparing cellulose-derivative-based hydrogels, temperature and pH of the dispersion are critical parameters, as the gelation mechanism of these polymers depends on temperature, and their optimal stability is pH-dependent. Thus, it is recommended to heat the macromolecule dispersion either before or after the polymer swelling.

**Figure 3:**
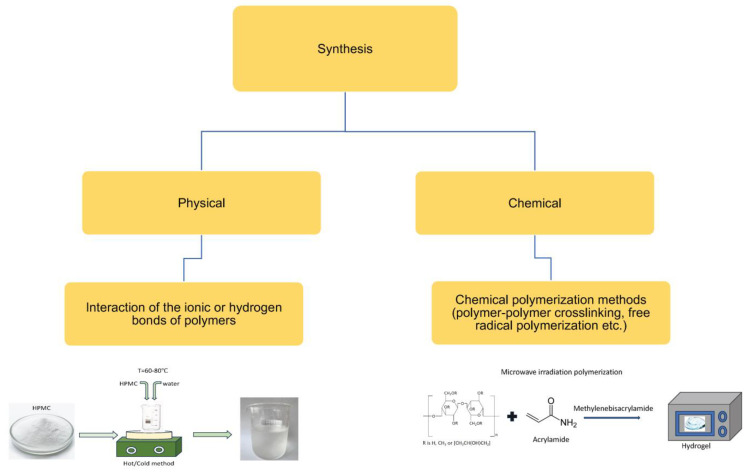
Method for preparing hydrogels based on HPMC [49,50].

Overall, the method for producing most hydrogels based on cellulose derivatives involves dispersing the polymer powder in cold water using mechanical stirring to form a homogeneous, single-phase dispersion. This is followed by heating the dispersion to approximately 60–80 °C and then gradually cooling it to room temperature to form the gel, a process known as the “hot/cold” method (Figure 3) [49,50]. Additionally, there are differences in the pH values of the dispersion medium favorable for gel formation: NaCMC, MC, and HPMC gels form over a wide pH range (4–10), while HPC and HEC form gels at a pH of 6–8 and under alkaline pH conditions, respectively. Another important parameter in the preparation of hydrogels from cellulose derivatives is the swelling duration of the polymer. Typically, a swelling time of about 24–48 h is required to obtain homogeneous gels.

Finally, the removal of entrapped air is also an important consideration in the preparation of HPMC hydrogels as the presence of air bubbles in the gel inevitably affects its transparency. The inclusion of air bubbles in the gel can be minimized by positioning the stirrer at the bottom of the mixing container. Typically, air bubbles are further removed using various methods, including prolonged storage, storage at low temperatures, ultrasonic treatment, or the addition of silicone-based defoamers. Additionally, in large-scale production, vacuum deaerators are used to remove entrapped air. In small-scale production, the preparation of hydrogels by manually or mechanically dispersing the polymer in hot/cold water or a co-solvent is carried out using simple equipment and utensils available in a pharmaceutical laboratory, such as porcelain or glass mortars and pestles, measuring cylinders, magnetic stirrers, and various propellers and mixers. In large-scale production of pharmaceutical cellulose derivatives, various mills, separators, mixers, deaerators, and switches are used. In the pharmaceutical application of HPMC gels, the physical method of production is the most convenient and popular, especially when creating medicinal systems with plant extracts.

## 4. Important Physicochemical Properties of HPMC and Its Hydrogels

HPMC (hydroxypropyl methylcellulose) is a modified propylene glycol ether of methylcellulose, consisting of methoxy and hydroxypropyl groups along the linear polysaccharide chain (*P. Timmins* [39]).

Cellulose derivatives, particularly HPMC, are among the widely used natural biodegradable polymers applied in the development of oral drug delivery systems with controlled release [51]. The variation in cellulose derivatives regarding molecular weight, solubility, viscosity, and degree of dissociation allows for their extensive use in various applications. The main property of HPMC, which is used in the creation of immobilized systems and in the mechanism of drug release, is its solubility and swelling of its gels, which allows for a variation in parameters to achieve different degrees of prolongation.

The physicochemical properties of HPMC, such as surface activity, solubility, and gel formation, depend on three factors: the presence of methoxy groups, the presence of hydroxypropyl groups, and molecular weight. HPMC is soluble in cold water due to the targeted alkylation of the hydroxyl group of natural cellulose with methoxy and hydroxypropyl groups [15]. After dissolving in water, it forms a clear viscous gel. Hypromellose also dissolves very well in other solvents, such as ethanol, isopropanol, dichloromethane, as well as in their aqueous solutions at various ratios.

Temperature significantly affects the properties of the gel formed from HPMC. Aqueous solutions of hypromellose exhibit sol-gel thermal transitions at high temperatures, which most often manifest as turbidity of the solution caused by the process of hydrophobic interaction between the methoxyl groups in the hypromellose [52]. For hypromellose, the gelation process begins with the dissolution of the polymer, and with a further increase in temperature, the formation of terminal separated sections and hydrophobic associations of chains occurs. Hydration of hypromellose is highly temperature-dependent; as the temperature increases, HPMC loses hydration water, which leads to a decrease in the relative viscosity of the polymer, and the loss of hydration water contributes to the strengthening of polymer–polymer interactions, such as hydrophobic ones, primarily due to methoxy substituents [53]. Increased hydrophobic interactions lead to increased viscosity, which is the point of gelation. The gelation temperature of hydroxypropyl methylcellulose (HPMC) ranges from 50 °C to 90 °C and depends, like the solidification temperature, on the number of methoxy groups in the molecule [54]. Substitution with ethoxy and dioxy groups contributes to an increase in the gelation temperature. The results of the study [55] showed that the gelation temperature can be reduced to 36 °C by replacing the hydroxypropoxy groups.

As the temperature increases, the gelation process occurs, leading to the precipitation of polymer molecules [56]. The gelation point and the turbidity point are independent of each other; so, the solution may become turbid before reaching the gelation temperature. If a polyelectrolyte is present in the solution, both the turbidity and the gelation temperature decrease [53].

HPMC is not used in materials that require thermal treatment because its decomposition temperature is 170–180 °C, and its melting point is 190–200 °C. Moreover, the decomposition temperature of HPMC depends on the structure of the side chains [57]. The lack of thermal stability prevents the use of further processing methods for the polymer that require its melting. Thus, in [58], attempts were made to improve the polymer’s thermal stability. The development by the chemical company “Dow” (USA) for the extrusion and spray drying process required the use of hot molten polymer. A new product under the brand name “Affinisol HPMC HME” has been proposed, which features a low glass transition temperature, low viscosity, color stability at high temperatures, and extrusion capability. The authors also achieved improved solubility in organic solvents, which allows the polymer to be used in spray drying.

The source of hydroxypropyl methylcellulose (HPMC) is cellulose fibers, which are heated with an acoustic alkaline solution during the etherification process [16]. It is then treated with propylene oxide and methyl chloride. The resulting product has a specific ratio of substituted hydroxypropyl and methyl groups, which, in turn, depends on the ratio of methyl chloride and propylene oxide introduced after alkalization. The ratio of substituted groups affects the viscosity and gelation temperature of aqueous solutions of hypromellose.

The glass transition temperature of the polymer plays an important role in the development of controlled drug delivery systems. It was previously mentioned that hypromellose is widely used as a filler in pharmaceutical formulations and for the development of controlled drug delivery systems. When taking drugs with controlled delivery, some properties affect the release of the active drug. For example, it is known that a decrease in the glass transition temperature of the polymer reduces the mobility of macromolecules within the structure. At the same time, as the temperature increases, the mobility of macromolecules begins to rise, leading to an increased rate of drug transport. In the work of Doelker E. et al. [59], the glass transition temperature of the HPMC polymer was investigated. Various methods, such as thermomechanical analysis, dynamic mechanical analysis, differential scanning calorimetry, and torsion analysis, were used to determine and compare the results. However, different analysis methods show different glass transition temperature results for HPMC. Therefore, it is recommended to conduct research and comparisons using one of the methods. In determining the glass transition temperature, it is important to consider the degree of substitution as well as the molecular weight of the polymer [15]. All the listed properties of HPMC are important in the creation of medicinal hydrogel forms. The nature and structure of the active pharmaceutical ingredient adjust its properties, especially rheological characteristics, swelling properties, solubility, compatibility, uniformity, and physico-mechanical strength.

Hydrogel systems with phytocompounds based on polysaccharides show improved properties such as biocompatibility, controlled release of active substances, mechanical strength, and hydrophilicity. Cellulose ethers have all of the above properties, and HPMC occupies a worthy place. The works summarize research on the production of polysaccharide hydrogels with phytocompounds in various dosage forms using solution casting technologies, with porogenic agents, gas foaming, lyophilization, cryogelation, extrusion, coacervation, emulsification, spray drying and electroforming [60,61]. In [62], cellulose and CMC are mentioned among the popular carriers, but it should be noted that, in recent years, there has been increasing interest in obtaining hydrogel systems of synthetic biologically active substances, phytocompounds with hypromellose (HPMC) [43,63,64,65,66,67,68,69,70,71,72]. The main focus in [65] is on properties such as thermosensitivity, swelling capacity, physical and mechanical properties, and the release of biologically active substances. The high swelling tendency, hydrophilicity, and thermal sensitivity of HPMC made it possible to use it to obtain a “smart” and biocompatible film with a uniform surface. The formation of hydrogen bonds between the NH- and OH-groups of chitosan and HPMC increases the strength, and the degree of swelling reaches the minimum value of the CH:HPMC 40:60 system. Due to chitosan, the hydrogel system acquires pH sensitivity and the phase transition temperature of HPMC increases. The introduction of essential oils into the composition of the CH-HPMC hydrogel increases the thixotropic and rheological properties, and enhances the penetration of fluconazole [66]. In the binary PVP-HPMC gel, improved adhesive properties and rheological characteristics are observed. Bases made from both pure HPMC and the binary PVP-HPMC mixture [43] contribute to the slow release of the model drug benzophenone-4. Additionally, the release of the active substance is further slowed by changes in viscosity, which significantly increases at concentrations above 8%. Upon contact with the skin and intense rubbing, viscosity rapidly decreases, ensuring gel spreadability. The addition of PVP enhances the prolongation of drug action due to the electrostatic interaction between PVP and benzophenone-4. An HPMC-based hydrogel [67] containing PGA microfibers and ofloxacin, formed through blending, exhibits high wound-healing activity, a swelling degree of 531.8–1700%, and elongation within the range of 70–120%. The synergistic antimicrobial effect of the microfibers and the active ingredient is enhanced by the strong swelling capacity of HPMC, which plays a crucial role in the tissue granulation stage and promotes rapid wound healing.

Studies have demonstrated the possibility of obtaining a porous HPMC gel containing carvedilol, dissolved in Tween-20, lactic acid [68], and polysaccharides from *Agaricus blazei Murill* [69], with controlled release and rheological properties suitable for bone tissue regeneration. The gelation temperature of HPMC was reduced to 37 °C due to the presence of polysaccharides, making it comfortable for the oral mucosa and dental restoration. Additionally, the porosity of the HPMC gel allowed for the regulation of the release of poorly soluble carvedilol. As is well known, the rheological characteristics, swelling properties of HPMC, and gelation temperature are essential for the development of pharmaceutical dosage forms, particularly prolonged-release tablets with controlled drug release [70,71,72].

Researchers have described the kinetics of release of the active drug substance from a controlled delivery system based on hypromellose [73,74]. Various methods of analysis have been used, such us NMR spectroscopy, to characterize the mobility of water in the gel layer of the HPMC polymer [75].

## 5. Relevance of Pharmaceutical Application of HPMC with BAS

According to the WHO, more than 80% of medicines are of plant origin. Most diseases require the use of medicinal herbs along with synthetic drugs. Enhancing the efficiency of plant extracts and the rational use of natural resources are among the pressing global issues included in the Sustainable Development Goals due to the growing number of patients with skin diseases, trophic ulcers, and purulent wounds of various etiologies, necessitating a range of affordable applicative materials and their production [76,77].

The growing interest in the field of natural compound chemistry research is explained by several factors:

(1) The diversity of both the chemical structures and biological activities of naturally occurring secondary metabolites;
(2) The use of new bioactive natural compounds as biochemical probes;
(3) The development of new and sensitive methods for detecting biologically active natural products;
(4) Improved isolation methods;
(5) Purification and structural characterization of these active components, as well as the demand for natural products [76].

The scientific literature includes studies on the development of wound-healing pharmaceutical formulations based on natural polymers and plant extracts [78,79]. The biological activity of medicinal plants and their extracts is determined by their components, which define their beneficial properties such as antioxidant, wound-healing, anti-inflammatory, antibacterial, anti-tumor, and other effects. Among the natural components of plants, phenolic compounds and secondary metabolites that exhibit the abovementioned activities stand out. An example of the application of natural polymers is the development of a wound-healing dosage form based on alginates. Leading positions in the creation of polymeric dosage forms are held by biopolymers that are biodegradable and typically derived from natural sources, such as plants, algae, animal organisms, and bacteria. Examples of such biopolymers include polysaccharides, lipopolysaccharides, and proteins, which hold a significant share of the pharmaceutical sector [80]. Thus, Klemm D. et al. [81] propose a range of affordable, biodegradable biopolymers with low cost, which can serve as the basis for medicinal products, particularly medical wound-healing dressings. Among biopolymers used as carriers for plant-based medicinal substances, HPMC stands out, as evidenced by data on HPMC-based materials combined with biologically active substances, extracts, and synthetic compounds, demonstrating enhanced effectiveness in wound treatment. Examples of HPMC applications as a drug carrier can be found in the works of many researchers. Below are some examples of soft dosage forms based on HPMC with medicinal substances and plant components (Table 1).

The analysis of studies and data presented in Table 1 shows that the geographic application of HPMC as a carrier of biologically active substances (BASs) is quite extensive. The advantageous properties of HPMC for the creation of pharmaceutical formulations enable the development of soft transdermal systems incorporating biologically active substances (BASs). Among the developed formulations, gel and film forms with HPMC are the most popular [65,66,67,68,69,70,71,72,73,74,75,76,77,78,79,80]. The studies on obtaining capsules, biomembranes, nanocrystals, and nanofibers are particularly interesting. The soft biomaterials developed are proposed as antibacterial, wound-healing, antimicrobial, and anti-inflammatory agents for the treatment of burns of varying degrees, as membranes, sorbent materials, and drug delivery carriers. As the data presented in the table show, HPMC plays an important role in hydrogel formulations, providing gelation, moisture retention, controlled release and prolongation of the action of medicinal components.

In the process of wound healing, plant extracts show activity and advantages due to the content of biologically active substances such as flavonoids, terpenoids, fatty acids, phenolic compounds, alkaloids, catechins, quinones and carbohydrates [106,107]. The main disadvantage of plant-based biologically active substances is their short duration of action and instability, which can be solved by the creation of hydrogel dressing materials based on natural polymers. Secondary metabolites of plants exhibit antibacterial, anti-inflammatory, antioxidant and regenerative effects. The stages of the physiological process of wound healing include platelet aggregation, the appearance of a hemostatic effect (hemostasis), the formation of macrophages that cleanse damaged tissue with further tissue growth (inflammatory phase), the stage of re-epithelialization and the formation of granulation tissue with the participation of fibroblasts, keratinocytes and endothelial cells (proliferative phase), and the stage of replacing type III collagen with type I collagen with strengthening of the formed tissue (remodeling). Plant extracts participate in stimulating the process of formation of new blood vessels, the flow of nutrients to damaged tissue, the removal of degradation products, and the formation of granulation tissue and collagen.

Among plant extracts, aloe vera [70,94], henna [83], curcuma [108], *Araucaria heterophylla* [109], and others are popular in wound treatment and healing. These medicinal plants are used in the preparation of dosage forms with cellulose ethers, and with HPMC for the treatment of wounds and burns with the properties necessary for treatment. For wound healing, the most commonly used compounds are flavonoids—rutin, quercetin, kaempferol, phenolic compounds and terpenoids—tannins, anthocyanins, ellagic acid, and alkaloids—berberine, embelin, etc.

The anti-inflammatory activity of plant extracts is due to the presence of flavonoids (curcumin, quercetin, quercetin-3,7-di-O-α-L-rhamnopyranoside, Epigallocatechin-3-gallate, 5-Hydroxy-7,8-dimethoxyflavanone, 6,7-Dimethoxycoumarin, liquiritigenin, isoflavone, etc.), terpenes (8-o-acetylharpagide, schizanol, stigmasterol, 7-B-hydroxysitosterol, marinoid D, maslinic acid, oleanolic acid, glycyrrhetinic acid etc.), essential oils (eugenol, linalool, camphor, borneol, thymol, citral, menthol, γ-terpinene, thymoquinone, carvone, α-terpineol, etc.), alkaloids (colchicine, sinomenine, capsaicin, berberine), and phenolic compounds (10-Shogaol, 4-hydroxybenzoic acid, cis-mellitoside, trans-mellitoside, salicin, ferulic acid, dihydromellitoside, etc.) [110].

Plant extracts can be used individually or in combination as antibacterial components of medicines with well-known antibacterial drugs, providing a synergistic effect [111]. The antimicrobial properties are due to the presence of natural compounds such as allicin, piperine, curcumin, eugenol, chlorogenic acid, quercetin, carvacrol, thymol, etc.

The mechanisms of action of plant secondary metabolites (PSMs) on microbial cells include disrupting the structure and function of the bacterial cytoplasmic membrane, preventing complex formation with membrane proteins, inhibiting enzyme synthesis, blocking the synthesis and functions of DNA and RNA, and increasing the coagulation of cytoplasmic components [112]. Antimicrobial action based on the destruction of the membrane of microbial cells is exhibited by flavonoids, terpenes and terpenoids, essential oils. For alkaloids, the literature describes effects on key stages of the pathogenesis process, such as inhibiting the production of staphylococcal α-hemolysin (e.g., capsaicin from *Capsium* L.).

It is important to note that the extraction of plant biologically active substances is carried out through the preparation of decoctions, infusions and alcohol tinctures. In the study [111] of the antibacterial activity of plant extracts using the dual reporter system Dualrep2, it was shown that alcoholic extracts exhibit greater activity compared to aqueous ones, and that alcoholic extracts are better obtained from dried raw materials than from fresh ones.

Medicinal systems based on HPMC and its compositions are obtained both with individual synthetic biologically active substances and on the basis of plant extracts individually and in combination with known drugs. The *Aloe vera* plant (*Aloe barbadensis*) is rich in polyphenolic compounds with antioxidant activity and is abundant in vitamins. The anthraquinones and saponins in aloe gel exhibit antimicrobial activity against Gram-positive bacteria such as *Shigella flexneri* and *Streptococcus pyogenes* [113]. The presence of various classes of natural compounds in *Aloe vera* has enabled its use in an HPMC-based gel as a burn treatment for skin wound healing [87]. *Aloe vera* gel is an excellent and accessible alternative to antimicrobial agents among plant-based sources with a rich composition of phytometabolites. In the wound-dressing material with *Aloe vera* gel, HPMC is included into the composite base as the main film-forming and moisture-retaining component [94]. The similarity of the nature of HPMC and the active compounds of *Aloe vera*, such as polysaccharides (glucomannans), glycoproteins, amino acids, flavonoids, provides the fibrous material with strength and heat resistance due to the formation of bonds between these components. HPMC also facilitates the dispersion of *Aloe vera* in the polymer mixture, thus ensuring its uniform distribution.

For the treatment of burn infections caused by Gram-positive bacteria such as *Staphylococcus aureus* and *Pseudomonas aeruginosa*, a composition based on cellulose ether derivatives, extracts of henna (*Lawsonia inermis* L.) and chamomile (*Matricaria chamomilla* L.) was proposed [83]. The antibacterial effect of the composition is due to terpenoids, phenolic compounds of the alcohol extract of chamomile, and the presence of such active components of henna as Lawson (2-hydroxy-1,4-naphthoquinone), tannins. Also, the presence of a powerful antioxidant gallic acid, flavonoids, mucilagin, and mannitol contributes to an important stage, skin healing, in the treatment of burn wounds. The base of HPMC and CMC provides controlled release of active components by a diffusion mechanism.

## 6. Conclusions

The main types of hydrogels of cellulose ethers and their methods of synthesizing, classification, uses and chemical properties of hydrogel forms with BAS are reviewed in this article. Hydrogels of cellulose ethers have become a popular material for thorough research and practical use in a variety of fields due to their capacity to absorb and hold large amounts of liquid and due to its economical availability. Cellulose-based products are the most eco-friendly and biodegradable; thus, as hydrogels, when they reach the aquatic environment, they have the unique ability to maintain a three-dimensional structure. Hydroxypropyl methylcellulose is a good material for drug transfer as tablets, ointments, gels, films with prolonged effect.

In the therapeutic treatment of wounds of various etiologies, antibacterial, anti-inflammatory and antioxidant effects are important for the active principle, which can be characteristic of both individual synthetic drugs and the BAC of plant extracts. In recent years, there has been a trend to search for natural sources of biologically active substances that meet the requirements for wound treatment and can be a worthy alternative to well-known drugs, especially antimicrobial agents, to which resistance has already developed. The advantage of using plant extracts is that phytocomponents can act at several stages in the wound-healing process, from hemostasis to remodeling, which is possible due to phytometabolites such as flavonoids, terpenes, tannins, polysaccharides, terpenoids, phenolic compounds, alkaloids, essential oils, saponins. The similarity in nature and structure between phytometabolites and cellulose ether derivatives makes them promising for the development of biocompatible, biodegradable, effective, and controlled wound dressings, gels, films, fibers, and membranes.

To sum up, hydrogels of hydroxypropyl methylcellulose with plant extracts with biological active substances are of great interest to researchers as regards biomedical applications in the form of ointments, gels, and films for local antibacterial, antioxidant activities. A review of the literature showed that there is currently insufficient research on the biodegradation of drug delivery systems based on cellulose derivatives with biologically active substances, which is a broad field for further research.

## Figures and Tables

**Table 1:** List of HPMC-based formulations with biologically active substances (BAS).

Carriers	Active Pharmaceutical Ingredient (Natural and Synthetic)	Type of Wound Dressing	Advantages and Application	References
HPMC/CH	Essential oil/Fluconazole	Hydrogel	Treatment of superficial fungal infections	[66]
HPMC/PG	Lidocaine and prilocaine	Film	A hydrophilic additive (propylene glycol), when used in a water-insoluble membrane, acts as a channelizer, and increases the rate of release of the drug.	[82]
HPMC	Lawsonia inermis (henna) and Matricaria chamomilla	Gel	Stability, good viscosity, uniformity, extrudability, good extract release, effectiveness in the treatment of burns.	[83]
	Liposomal farnesol	Gel	HPMC with farnesol in the ratios of 1:2 and 2:1 had a good effect in the treatment of burns of the third degree.	[84]
	Bacteriophage	Gel	The gel obtained at a temperature of 37 °C in a percentage of 3% HPMC was found to be stable, effective in the treatment of wounds with an antibacterial effect, especially against Klebsiella pneumoniae bacteria.	[85]
	CuNPs-licorice and phenytoin	Gel	It is effective in acute inflammation by suppressing JAK 3 inflammation and synthesis of type I procollagen.	[86]
	Honey and Aloe vera	Hydrogel	The hydrogel obtained in a percentage of 3% HPMC was found to be quite viscous when applied to the wound after a burn. The hydrogel showed good antibacterial activity on Klebsiella pneumoniae.	[87]
	Cefotaxime sodium	Hydrogel	After 4 h, the hydrogel containing 3 percent HPMC 400 had a strong spread ability and had released all of the medication content.	[88]
	Haruan/fusidic acid	Film	Elongation at break and water vapor permeability were acceptable in films containing 1% and 2% plasticizers, respectively.	[89]
	Epigallocatechin-3-gallate	Film	Tensile strength and water vapor barrier properties were improved.	[90]
	Coper nanoparticles	Film	Antibacterial activity that is appropriate.	[91]
	Ibuprofen	Nanocrystals	To make nanosuspensions of several polymers, microfluidization and sonication were used.	[92]
HPMC/Chitosan/Sodium alginate	Lidocaine chloride and polymyxin B sulphate	Bio membrane	Mechanical parameters (elasticity, tension, stiffness) and thickness are appropriate; in vivo, the material has a strong antibacterial action that aids tissue regeneration.	[93]
HPMC/PVA/PVP-I/PEG	Aloe vera (2%, 4%, 6%)	Fibers	Fibers containing 6% aloe vera were thinner and lacked beading, resulting in a larger porosity of the fibers.	[94]
HPMC/Poly(lactic acid)	Tetracycline hydrochloride	Nanofibers	Antimicrobial activity and high water sorption rate.	[95]
HPMC K100M/Gum Odina/Gelatin	Fluconazole and ofloxacin	Sponge	The gum Odina-HPMC K100M: gelatin (1:1) formulation cured chronic wounds with good physicochemical qualities and antibacterial activity.	[96]
Na-CMC/ HPMC Citric Acid	Tetracycline Methylene Blue	Hydrogel films	Inhibitory activity against pathogenic microbes *S. aureus* and *E. coli*	[97]
HPMC/AMPS	Loxoprofen sodium	Hydrogel	pH-sensitive hydrogel with controlled drug release, exhibiting intense release in alkaline environment.	[98]
HPMC/Gelatin	Lamontrigin	Hydrogel	Hydrogel-based material produced using 3D printing for pediatrics.	[99]
HPMC/alginate	Albumin	Hydrogel	Controlled release of albumin	[100]
HPMC/PEG	Fibrion, extract of B. mori silkworm cocoons	Film	It possesses stability, rapid swelling, and bioadhesiveness, suitable for transmucosal delivery.	[101]
Hydrofobic HPMC/alginate	Diclofenac	Capsules	Controlled release of diclofenac	[102]
HPMC	Tramadol Hydrochloride	Film	Hydrogel film, analgesic agent for peripheral neurotherapy.	[103]
HPMC/co-PAA-co-MAA	Insulin	Hydrogel	Hydrogel, rectal suppositories for regulating blood glucose levels, hypoglycemic effect.	[104]
HPMC/collagen/sesame oil	Diclofenac	Hydrogel, Emulgel, Bigele	The best properties for a diclofenac carrier are exhibited by a bigele.	[105]

## Abbreviations

The following abbreviations are used in this manuscript:

HPMC	Hydroxypropyl Methylcellulose
Na-CMC	Sodium Carboxymethyl Cellulose
PVP	Polyvinylpyrrolidone
MC	Methylcellulose
HPC	Hydroxypropyl Cellulose
HEC	Hydroxyethyl Cellulose
PAA	Polyacrylic Acid
